# Metabolic, inflammatory, and oxidative stress markers in women exposed to secondhand smoke

**DOI:** 10.7717/peerj.5758

**Published:** 2018-10-18

**Authors:** Mohd Hanaffi Siti Hajar, Sanip Zulkefli, Shaaban Juwita, Mohd Noor Norhayati, Mohd Yusoff Siti Suhaila, Aida Hanum Ghulam Rasool, Mohamed Yusoff Harmy

**Affiliations:** 1 Department of Family Medicine, Universiti Sains Malaysia, Health Campus, Kubang Kerian, Kelantan, Malaysia; 2 Central Research Laboratory, Universiti Sains Malaysia, Health Campus, Kubang Kerian, Kelantan, Malaysia; 3 Pharmacology Vascular Laboratory, School of Medical Sciences, Universiti Sains Malaysia, Kubang Kerian, Kelantan, Malaysia; 4 Faculty of Medicine and Health Sciences, Universiti Sultan Zainal Abidin, Kuala Terengganu, Terengganu, Malaysia

**Keywords:** Adiponectin, Secondhand smoke, Nonesterified fatty acids, Interleukin-6, Oxidized low density lipoprotein cholesterol, Insulin resistance, High-sensitivity C-reactive protein

## Abstract

**Background:**

Secondhand smoke (SHS) exposure has adverse effects on the cardiovascular system. This study aimed to determine the effects of SHS on the cardiovascular disease biomarkers, namely the metabolic, inflammatory, and oxidative stress markers in healthy adult women.

**Methods:**

This comparative cross-sectional study was conducted among healthy women. The cases included those women exposed to SHS, and the controls included those women not exposed to SHS. SHS exposure was defined as being exposed to SHS for at least 15 min for 2 days per week. Venous blood was taken to measure the metabolic markers (high molecular weight adiponectin, insulin level, insulin resistance, and nonesterified fatty acids), oxidative stress markers (oxidized low density lipoprotein cholesterol and 8-isoprostane), and inflammatory markers (high-sensitivity C-reactive protein and interleukin-6). A hair nicotine analysis was also performed. An analysis of covariance and a simple linear regression analysis were conducted.

**Results:**

There were 101 women in the SHS exposure group and 91 women in the non-SHS exposure group. The mean (with standard deviation) of the hair nicotine levels was significantly higher in the SHS exposure group when compared to the non-SHS exposure group [0.22 (0.62) vs. 0.04 (0.11) ng/mg; *P* = 0.009]. No significant differences were observed in the high molecular weight adiponectin, insulin and insulin resistance, nonesterified fatty acids, 8-isoprostane, oxidized low density lipoprotein cholesterol, interleukin-6, and high-sensitivity C-reactive protein between the two groups. The serum high molecular weight adiponectin was negatively associated with the insulin level and insulin resistance in the women exposed to SHS. However, no significant relationships were seen between the high molecular weight adiponectin and nonesterified fatty acids, 8-isoprostane, oxidized low density lipoprotein cholesterol, high-sensitivity C-reactive protein in the SHS group.

**Discussion:**

There were no significant differences in the metabolic, oxidative stress, and inflammatory markers between the SHS exposure and non-SHS exposure healthy women. A low serum level of high molecular weight adiponectin was associated with an increased insulin level and resistance in the women exposed to SHS.

## Introduction

Secondhand smoke (SHS), also referred to as environmental tobacco smoke or passive smoke, is produced from the side-stream smoke released into the environment from the burning end of a cigarette or other tobacco product, or from the main-stream smoke exhaled by smokers (WHO, 2010). Globally, about one-third of all adults are regularly exposed to tobacco smoke. In Malaysia, an estimated 33.9% (1.4 million) of the nonsmoker adults who worked indoors were exposed to SHS in their workplace over the past 30 days; while at home, the estimated prevalence of exposure was 27.9% (4.2 million) (Ministry of Health, 2011).

The chemical toxicity present in SHS can cause adverse effects on the cardiovascular system, leading to coronary heart disease in adults (Barnoya & Glantz, 2005). Adiponectin is a protein that plays roles in both lipid and glucose metabolism. It exists in three forms, including low molecular weight, medium molecular weight, and high molecular weight (HMW) adiponectin (Waki et al., 2003). Studies have suggested that HMW adiponectin could be the most biologically active form, and that it represents a better marker for metabolic syndrome and insulin resistance when compared to the total adiponectin. Low levels of adiponectin have been associated with several cardiovascular disease (CVD) risk factors, such as obesity and type II diabetes (Nayak et al., 2009). In children, there are mixed evidences for the association between SHS exposure and adiponectin. Nagel et al. (2009) reported low adiponectin concentrations with SHS exposure, while Groner et al. (2015) reported no association.

Insulin resistance is a state where body secretes insulin but does not use it properly (NIDDK, 2008). Insulin resistance always been noticed in CVD such as diabetes, metabolic syndrome, obesity and stroke (Ginsberg, 2000; McFarlane, Banerji & Sowers, 2001) and is associated with an increased CVD risk (McFarlane, Banerji & Sowers, 2001). In the general population, with a wide range of glucose levels, SHS exposure has been associated with higher insulin resistance (Henkin et al., 1999). Previous studies have extensively discussed the relationship between SHS and the incidence of diabetes (Hayashino et al., 2008; Houston et al., 2006; Kowall et al., 2010). In addition, nonesterified fatty acid (NEFA) is an important factor in in the modulation of insulin metabolism, and it has been shown to be positively correlated with insulin resistance (Eliasson et al., 1994). Increased NEFA levels have been associated with diabetes. Moreover, it has been revealed that smoking elevates the NEFA levels in active smokers (Kershbaum et al., 1961). However, there are currently no studies that have examined the effects of chronic SHS exposure on the NEFA levels.

The free radicals from tobacco smoke may promote reactive oxygen species activation, which enhances inflammation and, consequently, increases oxidative stress levels in individuals exposed to SHS (Burke & Fitzgerald, 2003). Measuring the 8-isoprostane or F2-isoprostane is the gold standard for assessing oxidative stress; 8-isoprostane is derived from arachidonic acid via the process of lipid peroxidation. Another important oxidative stress marker that plays a significant role in the pathogenesis of atherosclerosis is oxidized low density lipoprotein cholesterol (LDL-C) (Yoshida & Kisugi, 2010). The oxidation of LDL-C from its native form generates the oxidized form known as oxidized LDL-C. It has been reported that high doses of SHS exposure increase the levels of oxidized LDL-C and 8-isoprostane in nonsmokers (Kato et al., 2006; Panagiotakos et al., 2004).

Inflammation mediates the formation and progression of atherosclerosis in the circulatory system. Chronic, high dose SHS exposure has been shown to be associated with increased inflammatory markers among adult nonsmokers (Panagiotakos et al., 2004). Moreover, metabolic changes, increased oxidative stress, and inflammation all contribute to increase the CVD risk. In addition, many SHS-exposed individuals have been reported to already exhibit increased CVD risk factors, such as type 2 mellitus (DeFronzo & Ferrannini, 1991).

A study simultaneously measuring metabolic, inflammatory and oxidative stress on the CVD risk in those exposed to SHS would provide valuable information. This study aimed to compare the effects of SHS on the CVD biomarkers (metabolism, oxidative stress, and inflammation) between women with and without SHS exposure. In addition, it aimed to determine the relationships between HMW adiponectin and insulin, insulin resistance, NEFA, 8-isoprostane, oxidized LDL-C, high sensitivity C-reactive protein (hsCRP) in women with SHS exposure.

In this study, The SHS exposure was defined as of at least 15 min in 2 days within a week exposure (WHO, 1986). Nearly similar definition has been applied in several epidemiological studies (Nor et al., 2008; Panagiotakos et al., 2004; Samet & Yang, 2001; Yang et al., 1999; Yang et al., 2010). In China they demonstrated that SHS exposure for at least 15 min daily for more than one day every week were well-correlated with hair nicotine concentration among women exposed to SHS at home (Yang et al., 2010).

## Methodology

### Population and sample

A comparative cross-sectional study was performed. The cases were defined as those women with SHS exposure, and the controls were those women without SHS exposure. The subjects who were exposed to tobacco smoke for at least 15 min for two days in one week were considered to have SHS exposure, while the non-SHS exposure group included women who were free from tobacco smoke (WHO, 1986). The SHS exposure was self-reported. The subjects were recruited from the staff of several government agencies in Kota Bharu, Kelantan, Malaysia. Women between 18 and 60 years old, without CVD histories, with body mass indexes (BMIs) <35 kg/m^2^, and who were not pregnant were included in this study. Those subjects who were current or former smokers, who had histories of hypertension, CVD, inflammatory disease, or other chronic disease, with total cholesterol level >6.5 mmol/l or fasting capillary plasma glucose ≥5.6 mmol/l, or who were taking lipid lowering or diabetic medications were excluded from this research.

A convenience sampling method was applied, and the sample size was calculated based on the comparison of two means using the PS: Power and Sample Size Calculation software (Dupont & Plummer Jr, 1998). Based on the standard deviation of the homeostatic model assessment of insulin resistance (HOMA-IR) of 1.6 (Nakashima et al., 2006), an alpha of 0.05, and a power of 0.8, the minimal required sample size was 87. After considering a nonresponse rate of 10%, the sample size calculated for each group was 96.

### Data collection

The protocol for this research project was approved by the Human Research Ethics Committee of Universiti Sains Malaysia (USM/PPP/JEPeM [255.3.(6)]), where this work was undertaken. During the subject screening, the blood pressure and pulse rate were measured using a digital blood pressure monitor (MX3; Omron, Kyoto, Japan). The blood pressure monitor cuff was applied to the subject’s right arm while she was in a sitting position, and the subject was given at least 10 min to rest before taking the blood pressure measurement. Two readings were taken at five minutes apart, and the mean reading was recorded. Those subjects with a seated systolic blood pressure ≥140 mmHg and diastolic blood pressure ≥90 mmHg were excluded from this study.

Based on their SHS exposure, the subjects were divided into case and control groups, and each underwent similar data collection procedures. An appointment was given and the subjects were requested to fast for 12 h prior. For the biochemical index measurements, 12 ml of the peripheral blood was collected from each subject. The serum concentration of the HMW adiponectin was measured using an enzyme-linked immunosorbent assay (ELISA) kit (MilliporeSigma, Burlington, MA, USA). The serum insulin was measured via the chemiluminescence method using an Elecsys Insulin kit (Roche Diagnostics, Mannheim, Germany). The insulin resistance was calculated using the HOMA-IR [HOMA-insulin resistance = (fasting glucose × fasting insulin)/22.5]. The serum NEFA levels were assayed using the enzymatic colorimetric method with a commercially available kit (Wako Pure Chemical Industries, Ltd., Osaka, Japan). The 8-isoprostane was measured from the serum samples using a competitive enzyme immunoassay (EIA) kit (Cayman Chemical, Ann Arbor, MI, USA). The serum oxidized LDL-C and interleukin-6 (IL-6) levels were determined using commercial ELISA kits (Cusabio Biotechnology Co., Ltd., Wuhan, Hubei Province, China and eBioscience, Inc., San Diego, CA, USA).

Blood for fasting lipid profile (FLP), HMW adiponectin, 8-isoprostane, insulin and NEFA were collected in 6 ml plain tube (BD Vacutainer®, Franklin Lakes, NJ, USA) with clot activator. Meanwhile, blood for full blood count (FBC) and fasting blood sugar (FBS) were collected in three ml EDTA tube and three ml potassium oxalate and sodium fluoride tube, respectively. FLP, FBC, FBS and hsCRP were analyzed on the same day as blood collection. Serum for HMW adiponectin, 8-isoprostane, insulin, oxidized LDL, hsCRP and NEFA were prepared by immediately centrifuge at 4,000 rpm for 15 min at 4 °C. Serum was aliquoted into five to eight micro-centrifuge tubes to avoid freeze thaw cycles. All tubes were labelled corresponding to each subject and stored immediately at −80 °C. Frozen samples were thawed at room temperature prior to assay.

In addition to the tests conducted above, approximately 15–20 strands of scalp hair measuring less than 10 cm each were cut and marked with tape. Each labelled sample was kept in an envelope and stored in an air-tight container at room temperature. Then, the hair was minced into approximately one mm pieces and placed in a test tube for washing. Deionized water was added to each test tube containing a hair sample until it was submerged, and the test tube was vortexed for 5 min. The deionized water was then carefully removed. Next, dichloromethane was added to each test tube until the sample was submerged, and it was vortexed for 5 min. Then, the hair sample was sonicated for 10 min before removing the dichloromethane solvent. The dichloromethane wash was repeated twice. Finally, the clean hair was air dried overnight in an open petri dish at 37 °C. The prepared samples were extracted and analyzed at the National Poison Center to determine the hair nicotine concentration using gas chromatography-mass spectrometry.

Hair nicotine measurement was performed using a gas chromatograph with mass spectrometer and auto sampler (Agilent Technologies, Santa Clara, CA, USA) in selected ion monitoring and split less modes. One µL injection volume of sample was used and nicotine was separated by a fused-silica capillary column, HP-5ms (30 m × 0.25 mm, i.d.0.25 µm film thickness; Agilent Technologies, USA) with helium flow at constant pressure. The temperatures for the injector and interface were set at 250 °C and 280 °C respectively while ionizing voltage was 70 eV. The GC oven separation temperature was programmed from 70 °C to 230 °C (3 min hold) at a rate of 25 °C/min. Quantifying ions used in SIM mode were m/z 162 for nicotine and m/z 169 for internal standard (Man et al., 2009)’.

### Statistical analysis

The statistical analysis was performed using IBM SPSS Statistics for Windows, version 22.0 (IBM Corp, Armonk, NY, USA). Descriptive independent-*t* and chi-squared analyses were used. An analysis of covariance was performed to determine the differences in the metabolic, oxidative stress, and inflammatory markers between the SHS and non-SHS exposure groups after controlling for the age, BMI, triglycerides, and HOMA-IR. A simple linear regression analysis was used to determine the strength of the associations between the HMW adiponectin and the metabolic markers (insulin, HOMA-IR, and NEFA), oxidative stress markers (oxidized LDL-C and 8-isoprostane) and inflammatory markers (hsCRP and IL-6) in the SHS exposure group.

## Results

Among the 250 women screened, only 192 subjects were eligible for this study. Table 1 shows the sociodemographic and clinical characteristic of the SHS and non-SHS exposure groups. The education level of the women in the SHS exposure group was significantly lower when compared to the non-SHS exposure group.

**Table 1:** Sociodemographic and clinical characteristics between SHS (*n* = 101) and non-SHS (*n* = 91) groups.

Variable	Second-hand smoke		Non-second-hand smoke		*P*-value^b^
	Mean (SD^a^)	*n* (%)	Mean (SD^a^)	*n* (%)	
***Sociodemographic***					
Age (years)	33.8 (7.86)		35.2 (9.84)		0.258
Ethnicity					
Malay		94 (53.4)		82 (46.6)	0.459^c^
Non-Malay		7 (43.8)		9 (56.2)	
Occupation					
Government servant		59 (57.3)		44 (42.7)	0.080^c^
Private sector		11 (61.1)		7 (38.9)	
Housewife		24 (51.1)		23 (48.9)	
Others		7 (29.2)		17 (70.8)	
Education					
Primary & secondary		75 (59.5)		51 (40.5)	0.008^c^
College & university		26 (39.4)		40 (60.6)	
***Clinical***					
SBP (mmHg)	114.9 (11.85)		114.4 (11.55)		0.776
DBP (mmHg)	72.6 (8.94)		72.9 (8.85)		0.790
BMI (kg/m^2^)	24.9 (4.71)		24.3 (4.05)		0.320
Waist (cm)	76.4 (10.75)		74.6 (9.01)		0.211
FBS (mmol/L)	5.7 (0.68)		5.7 (0.71)		0.785
HDL (mmol/L)	1.7 (0.37)		1.7 (0.34)		0.178
LDL (mmol/L)	3.3 (0.79)		3.2 (0.82)		0.676
TG (mmol/L)	1.0 (0.39)		1.2 (0.52)		0.068
TWC (×10$\hat {}$9/L)	6.8 (1.96)		6.4 (1.72)		0.150
Hematocrit (L)	0.4 (0.03)		0.4 (0.03)		0.393
Hb (gm/L)	131.0 (11.45)		129.1 (11.47)		0.236
Platelet (×10$\hat {}$9/L)	281.2 (57.73)		279.4 (69.78)		0.844

**Notes.**

a Standard deviation.

b Independent *t* test.

c Chi squared test.

SBP systolic blood pressure DBP diastolic blood pressure BMI body mass index FBS fasting blood sugar HDL high density lipoprotein LDL low density lipoprotein TG triglycerides TWC total white count Hb haemoglobin

The mean (with standard deviation) hair nicotine concentration in the SHS group was significantly higher when compared to the non-SHS group (0.22 (0.62) vs. 0.04 (0.11) ng/mg; *P* = 0.009). The nicotine levels in SHS and non-SHS groups ranged from 0 to 4.41 ng/mg and 0 to 0.90 ng/mg, respectively.

Table 2 shows the metabolic, oxidative stress, and inflammatory markers in the SHS and non-SHS exposure groups, and there were no significant differences between the groups. Table 3 shows that the serum HMW adiponectin was negatively associated with the insulin level and insulin resistance (HOMA-IR) among the women with SHS exposure. Among women exposed to secondhand smoke, each 1 mU/L increase in insulin was associated with a 0.29 mg/mL decrease in HMW adiponectin (95% CI [−0.40, −0.17]; *p* < 0.01) and each one unit increase in HOMA-IR was associated with a 1.05 unit decrease in HMW adiponectin (95% CI [−1.47, −0.63]; *p* < 0.01). No significant associations were seen between the HMW adiponectin and the NEFA, oxidized LDL, 8-isoprostane, hsCRP, and IL-6.

**Table 2:** Metabolic, oxidative stress and inflammatory markers in SHS and non-SHS exposure groups.

Variable	Second-hand smoke	Non-second-hand smoke	*P*-value^b^
	Mean (SD^a^)	Mean (SD^a^)	
***Metabolic***			
HMW adiponectin (µg/mL)	3.64 (2.28)	3.75 (2.63)	0.752
Insulin (mU/L)	8.25 (3.96)	8.27 (3.85)	0.980
HOMA-IR	2.12 (1.10)	2.07 (1.00)	0.757
NEFA (mmol/L)	0.85 (0.27)	0.81 (0.26)	0.394
***Oxidative stress***			
Oxidized LDL-C (mU/mL)	52.52 (22.74)	51.77 (22.55)	0.827
8-isoprostane (pg/mL)	279.21 (211.96)	275.00 (207.16)	0.934
***Inflammatory***			
hsCRP (mg/dL)	0.27 (0.30)	0.23 (0.25)	0.356
IL-6 (pg/mL)	4.00 (2.62)	3.61 (2.85)	0.324

**Notes.**

a Standard deviation.

b Independent *t* test.

HMW high molecular weight HOMA-IR Homeostatic model assessment of insulin resistance NEFA non-esterified fatty acid LDL low density lipoprotein hsCRP highsensitivity C-reactive protein IL-6 interleukin-6

**Table 3:** Association between HMW adiponectin and metabolic, oxidative stress and inflammatory markers in SHS exposure group using simple linear regression analysis.

Variable	HMW adiponectin		*P* value^a^
	b^a^ (95% CI^b^)	*R*^2^^c^	
***Metabolic***			
Insulin (mU/L)	−0.29 (−0.40, −0.17)	0.241	<0.001
HOMA-IR	−1.05 (−1.47, −0.63)	0.250	<0.001
NEFA (mmol/L)	−0.87 (−2.70, 0.96)	0.010	0.345
***Oxidative stress***			
Oxidized LDL-C (mU/mL)	0.00 (−0.02, 0.02)	0.000	0.850
8-isoprostane (pg/mL)	0.00 (−0.00, 0.01)	0.052	0.208
***Inflammatory***			
hsCRP (mg/dL)	−0.56 (−2.17, 1.04)	0.006	0.488
IL-6 (pg/mL)	−0.11 (−0.26, 0.04)	0.014	0.154

**Notes.**

a Regression coefficient.

b Confidence interval.

c Coefficient of determination.

HOMA-IR Homeostatic model assessment of insulin resistance NEFA non-esterified fatty acid LDL low density lipoprotein hsCRP high-sensitivity C-reactive protein IL6 interleukin-6

Based on self-reports, the nicotine concentration in SHS (*n* = 94) and non-SHS (*n* = 85) groups ranged from 0 to 4.41 ng/mg and 0 to 0.90 ng/mg, respectively. Whereas, based on hair nicotine, the nicotine concentration in SHS and non-SHS groups ranged from 0 to 4.41 ng/mg and 0 to 0.20 ng/mg, respectively. The number of subjects based on hair nicotine levels, and on self-report are shown in Table 4. Cross tabulation of the results showed significant agreement in classification by self-report and hair nicotine levels (*p* = 0.004), with agreement analysis-Cohen’s kappa coefficient (*κ*) value of 0.21 (Table 5).

**Table 4:** Number of subjects based on self-report and hair nicotine levels.

	Number of subjects in SHS	Number of subjects in non-SHS
Self-report	101 (>15 min exposure)	91 (<15 min exposure)
Hair nicotine^a^	81 (>LOQ^b^)	98 (<LOQ^b^)

**Notes.**

a Thirteen hair nicotine data cannot be retrieved.

b Limit of quantitation was 0.04 ng/mg.

**Table 5:** Cross-tabulation between self-report and hair nicotine.

		Hair nicotine		
		Non-SHS	SHS	Total
Self-report	Non-SHS	56	29	85
	SHS	42	52	94
	Total	98	81	179

**Notes.**

*κ* = 0.21, *P* = 0.004. Significant agreement between self-report and hair nicotine.

## Discussion

The SHS exposure for a minimum of 15 min for two days per week had no effects on the metabolic, oxidative stress, and inflammatory markers of the healthy adult women. However, an inverse association was seen between the serum HWM adiponectin and the insulin levels and insulin resistance among the women with SHS exposure. The education level of SHS group was significantly lower than non-SHS group. Similar finding was reported (Vitoria, Nunes & Precioso, 2017) and these confirmed the social inequalities associated with smoking.

Increased hair nicotine concentrations at 0.22 ng/mg were seen in the present study among the women in the SHS exposure group, which was slightly lower at 0.44 ng/mg in a prior study (Kim et al., 2009). The hair nicotine level range was within the ranges previously reported in women from various countries (0.02–2.5 ng/mg) (Wipfli et al., 2008). The nicotine that accumulates in growing hair reflects the long term exposure to SHS, in which 1 cm would represent 1 month of exposure. It has been reported that hair nicotine levels show better precision in defining the SHS exposure when compared to urine cotinine (Al-Delaimy, 2002).

This study did not find any significant differences in the HOMA-IR and NEFA levels between the two groups. Similarly, one study among the elderly population found no differences in the HOMA-IR and insulin levels between SHS and non-SHS exposure groups (Kowall et al., 2010). The absence of a difference in the healthy individuals may be because they are less susceptible to metabolic alterations, such as insulin resistance. One study demonstrated that the administration of nicotine intravenously reduced the insulin sensitivity in diabetic patients, but not in healthy nonsmokers (Axelsson et al., 2001).

Acute and chronic high dose SHS exposure has been shown to elevate the levels of 8-isoprostane (Ahmadzadehfar et al., 2006; Kato et al., 2006) and oxidized LDL (Panagiotakos et al., 2004). Therefore, the increase in oxidative stress may be more significant in short-term and intensive SHS exposure when compared to continuous long-term low doses of SHS exposure. The inadequate exposure to SHS in this study may have only depleted the antioxidant enzyme agents in the body, without causing any adverse effects on the LDL-C levels. It has been shown previously that the oxidative stress increased within the first hour after acute cigarette smoking, and it returned to normal within 90 min (Van der Vaart et al., 2004). Moreover, subjects with certain clinical characteristics, such as hypercholesterolemia and dyslipidemia, may have a higher probability of an oxidized LDL-C increase when compared to normal subjects (Holvoet et al., 2001).

The current inflammatory marker findings agree with a few recent reports observing acute short-term SHS exposure. Among healthy nonsmoking men, the hsCRP and IL-6 levels were found to be unaffected by SHS exposure (Bonetti et al., 2011), and the CRP concentrations did not differ between the smokers and nonsmokers (Abbasi et al., 2006). In addition, the CRP concentrations were not related to the adiponectin in either group. In contrast, one study reported that the CRP levels were higher in the SHS exposure group (Panagiotakos et al., 2004). This difference might be explained by the SHS exposure time. The minimum exposure time for this study was set at 15 min per day, while the others were set at 30 min per day. This limited SHS exposure may have failed to elicit significant inflammatory activation in the subjects of the current study.

Currently, there is no accepted, defined normal range of HMW adiponectin in adults. The values vary in studies. HMW adiponectin levels in our study were 3.64 (2.28) µg/ml in SHS group and 3.75 (2.63) µg/ml in non-SHS group. HMW adiponectin levels in healthy subjects from previous literature have ranged between 2.66 and 4.71 ug/ml (Goto et al., 2014; Nien et al., 2007; Zhu et al., 2010; Ziai et al., 2011). We found significant negative association between the HMW adiponectin levels and HOMA-IR and insulin levels in the SHS exposure group with correlation coefficient (B) were −1.05 and −0.29, respectively. Meaning each 0.29 µg/mL decrease in HMW adiponectin was associated with 1 mU/L increase in insulin and each 1.05 unit decrease in HMW adiponectin was associated with a 1 unit increase in HOMA-IR. In a study among Japanese, the authors found that HMW adiponectin was inversely associated with HOMA-IR with correlation coefficient (B) of −0.24 which means a unit of HOMA-IR was increased with the decrease of 0.24 µg/mL of HMW adiponectin. In short, decrease in HMW adiponectin is associated with diabetes. Goto et al. (2014) also found significant lower levels of HMW adiponectin in smokers compared to non-smokers with mean difference of 1.2 ug/ml.

Previous study has reported a negative association (*r* =  − 0.279, *P* < 0.001) between HMW adiponectin and HOMA-IR in smokers (Kawamoto et al., 2010). Meanwhile, the association between smoking and adiponectin in smokers has been reviewed and it was found that adiponectin levels were decreased in smokers compared to non-smokers (Kotani et al., 2012). The review also reported the significant relationship between smoking and adiponectin were unaffected even after adjusting the obesity-related traits in the analyses. The authors suggested that both nicotine and smoking-induced pathway may directly and indirectly be associated with low adiponectin levels in smokers. Briefly, smoking or exposure to cigarette smoke may decreased the adiponectin levels, thus, increased the insulin resistance and insulin levels, and this situation will increased the risk of diabetes mellitus in smokers and SHS exposure group.

In one community-based cross-sectional study, the serum HMW adiponectin was negatively correlated with the HOMA-IR among healthy adult male subjects; therefore, it was suggested to be useful for evaluating the presence of insulin resistance and metabolic syndrome in healthy populations (Seino et al., 2007). Moreover, adiponectin is an important modulator for insulin sensitivity. Among Pima Indians (Native Americans living in central and southern Arizona), the adiponectin was positively associated with the insulin sensitivity, and low concentrations of circulating adiponectin predicted a future decrease in the insulin sensitivity (Stefan et al., 2002). Smoking decreases the adiponectin levels, and this may be caused by the presence of the nicotine and oxidative stress promoters in tobacco, which reduce the mRNA expression and adiponectin secretion of adipocytes (Iwashima et al., 2005). Smoking, adiponectin, and insulin sensitivity are related in that smoking has the potential to reduce the adiponectin concentrations, and consequently, it leads to reduced insulin sensitivity in smokers, a phenomenon perhaps similarly seen in subjects exposed to high levels of SHS.

The strength of this study lies in the measurement of hair nicotine levels. However, there were a few limitations. For example, several important determinants of the HWM adiponectin, such as the body fat content, were not measured. One previous study showed the presence of a strong relationship between the adiponectin and the body fat percentage (Smith et al., 2006). Third, the presence of high antioxidants in some foods may influence the effects of SHS exposure by reducing the oxidative stress. However, the dietary habits of subjects in this study were not explored. Fourth, the SHS exposure was self-reported, and this may have introduced recall bias. Finally, this study was a cross-sectional study; therefore, the causal relationship cannot be determined.

## Conclusion

The SHS exposure did not change the metabolic, oxidative stress, and inflammatory marker profiles significantly among the healthy adult women. Nevertheless, a low serum HMW adiponectin level was inversely associated with an increased insulin level and resistance, which proved the harmful effect of SHS exposure on insulin regulation. We recommend future studies of the effects of SHS exposure based on objective assessment in other populations, such as male adults, with follow up in prospective cohort studies.

##  Supplemental Information

10.7717/peerj.5758/supp-1 Data S1 Raw data

## Additional Information and Declarations

### Data Availability

The following information was supplied regarding data availability: Raw data are provided in the Data S1.
